# Supplementary material for: R1441C and G2019S LRRK2 knockin mice have distinct striatal molecular, physiological, and behavioral alterations
Source: Commun Biol. 2022 Nov 10;5:1211. doi: 10.1038/s42003-022-04136-8 (PMC9649688; doi:10.1038/s42003-022-04136-8)
Supplement: Supplementary file 2 — Supplementary Information [file 42003_2022_4136_MOESM2_ESM.pdf]

R1441C and G2019S LRRK2 knockin mice have distinct striatal molecular, physiological, and behavioral alterations

Harry S. Xenias[1], Chuyu Chen[2*], Shuo Kang[2], Suraj Cherian[1], Xiaolei Situ[2], Bharanidharan Shanmugasundaram[2], Guoxiang Liu[3], Giuseppe Scesa[2], C. Savio Chan[1#], Loukia Parisiadou[2#]

[1] Department of Neuroscience, Feinberg School of Medicine, Northwestern University, Chicago, IL, USA

[2] Department of Pharmacology, Feinberg School of Medicine, Northwestern University, Chicago, IL, USA

[3] Department of Neurology, Feinberg School of Medicine, Northwestern University, Chicago, IL, USA

equal contribution

[#]corresponding authors

Correspondence should be addressed to C. Savio Chan at saviochan@gmail.com or Loukia Parisiadou at loukia.parisiadou@northwestern.edu

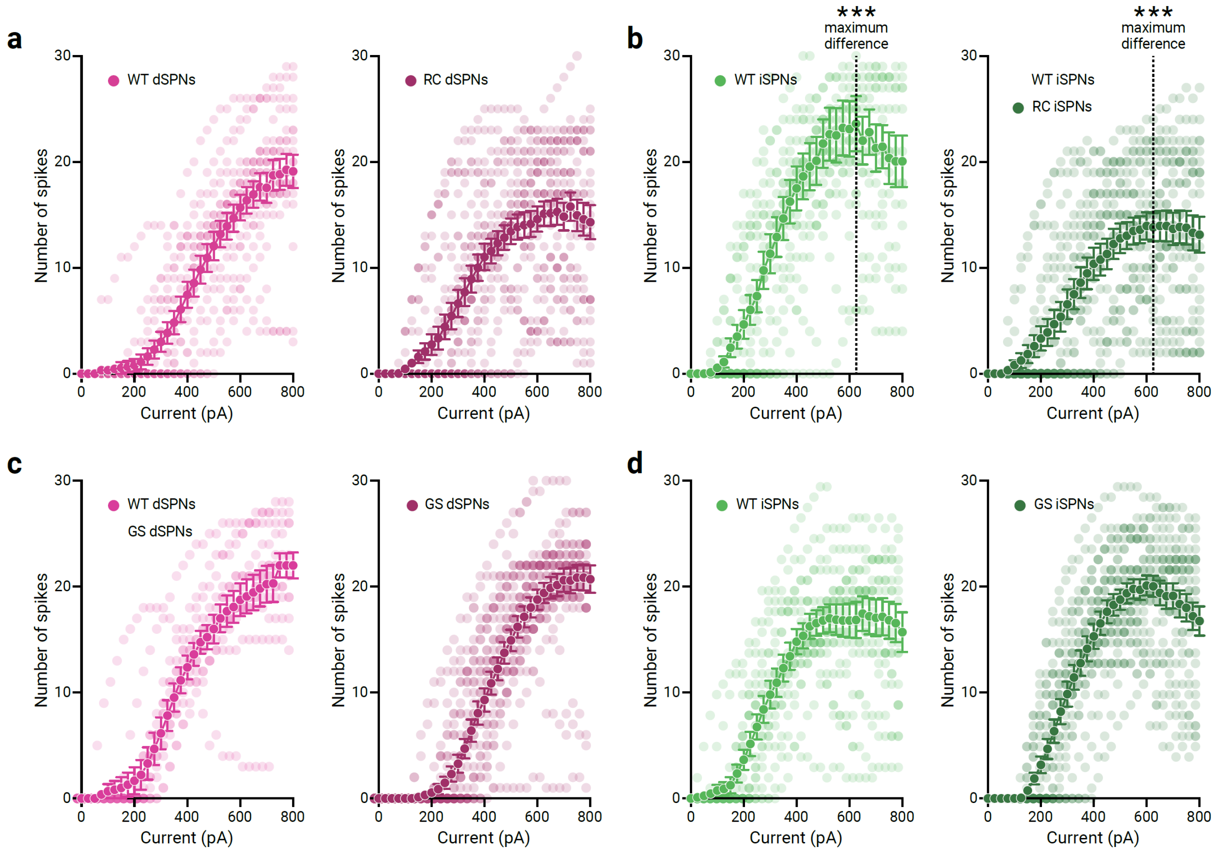

Supplementary Figure. 1. iSPNs in RC LRRK2 mice have decreased excitability.

Same as Figure 1 with individual data points shown.

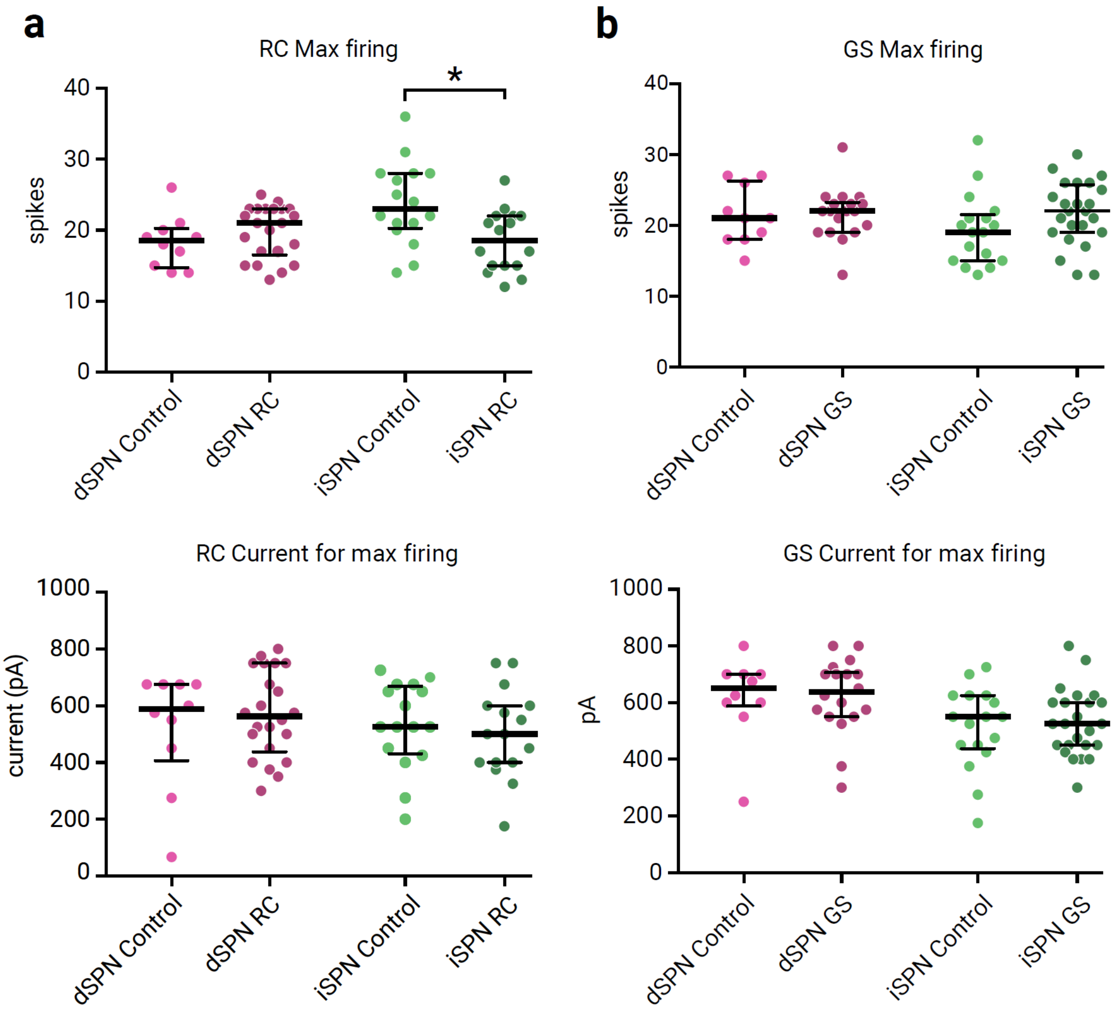

Supplementary Figure 2. Maximal firing across sweeps.
RC versus WT maximal firing values across recording sweeps per cell restricted to the injection range of 0–800 pA. b. GS versus WT maximal firing values across recording sweeps per cell are restricted to the injection range of 0–800 pA. Only for iSPNs in RC mice were there differences, where only the maximum firing was decreased ($spikes_{WT}$ = 23.0 ± 4.5, n = 16 cells; $spikes_{RC}$ = 18.5.0 ± 3.5, n = 16 cells; p = 0.0114, Mann–Whitney U test). *denotes $p < 0.05$. See Supplementary Table 3 for complete sample sizes and statistical results. *denotes $p < 0.05$.

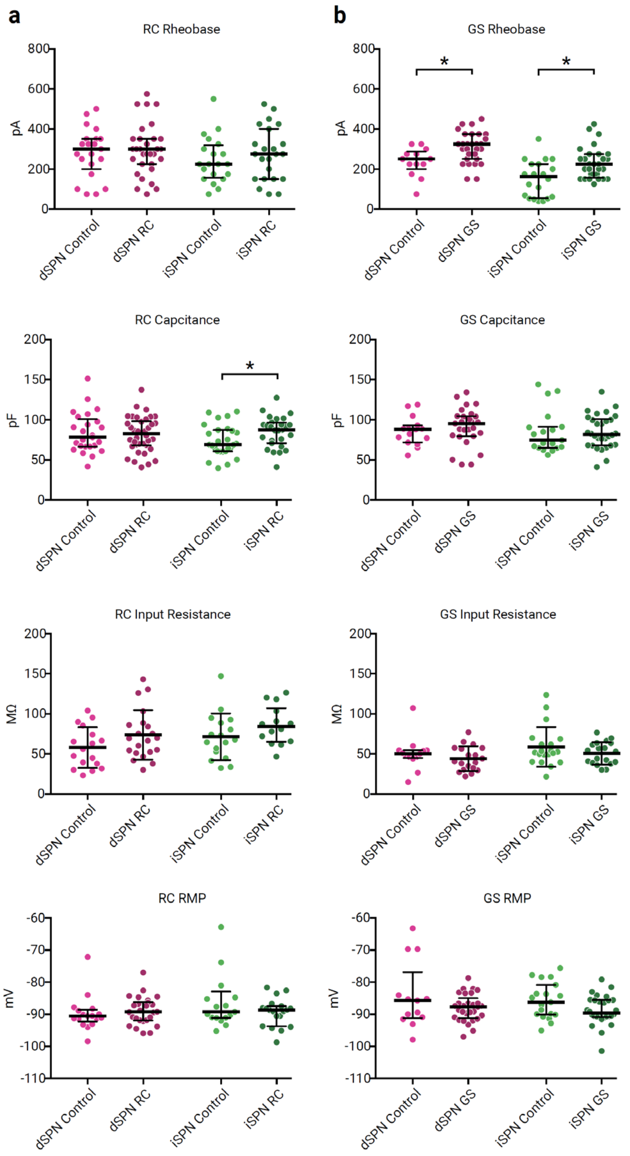

Supplementary Figure 3. General membrane electrophysiological properties. a. General membrane properties for the rheobase, capacitance, input resistances, and resting membrane potential in RC and WT mice. The membrane capacitance in RC mice was increased for iSPNs(Cm_{WT} = 68.83 ± 13.89, n = 27 cells; Cm_{RC} = 87.38 ± 13.34, n = 27 cells; p = 0.0388, Mann–Whitney U test). **b**. General GS versus WT passive membrane properties. The rheobase in RC mice was increased for both dSPNs ($rheobase_{WT}$ = 250 ± 25, n = 13 cells; $rheobase_{RC}$ = 325 ± 50, n = 29 cells; p = 0.0078, Mann–Whitney U test) and iSPNs ($rheobase_{WT}$ = 162.5 ± 87, n = 20 cells; $rheobase_{RC}$ = 225 ± 50, n = 28 cells; p = 0.0046, Mann–Whitney U test). * denotes $p < 0.05$. See Supplementary Table 4 for complete sample sizes and statistical results.

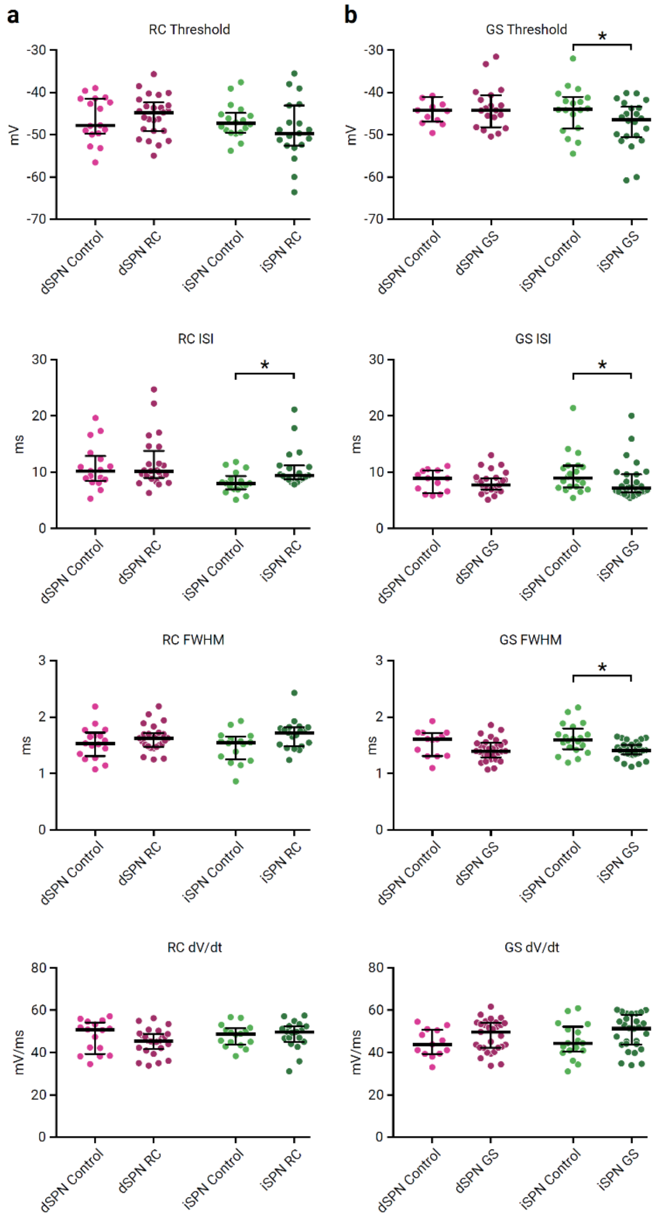

Supplementary figure 4. Action potential characteristics. a.RC versus WT spike threshold, interspike interval (ISI), full width at half maximum (FWHM), and rate of change of voltage after action potential depolarization. The ISI of iSPNs in RC mice were increased (ISI_{WT} = 8.0 ± 1.0 ms, n = 17 cells; ISI_{RC} = 9.4 ± 1.2, n = 19 cells; p = 0.0020, Mann–Whitney U test). b. GS versus WT spike threshold, interspike interval (ISI), full width at half maximum, and rate of change of voltage after action potential depolarization (dV/dt). The ISI of iSPNs in GS mice were decreased (ISI_{GS} = 8.95 ± 1.85 ms, n = 20 cells; ISI_{GS} = 7.15 ± 1.05, n = 28 cells; p = 0.0401, Mann–Whitney U test). The FWHM of iSPNs in GS mice were increased ($FWHM_{GS}$ = 1.54 ± 0.14, n = 16 cells; $FWHM_{GS}$ = 1.72 ± 0.11, n = 19 cells; p = 0.0039, Mann–Whitney U test). * denotes $p < 0.05$. See Supplementary Table 5 for complete sample sizes and statistical results.

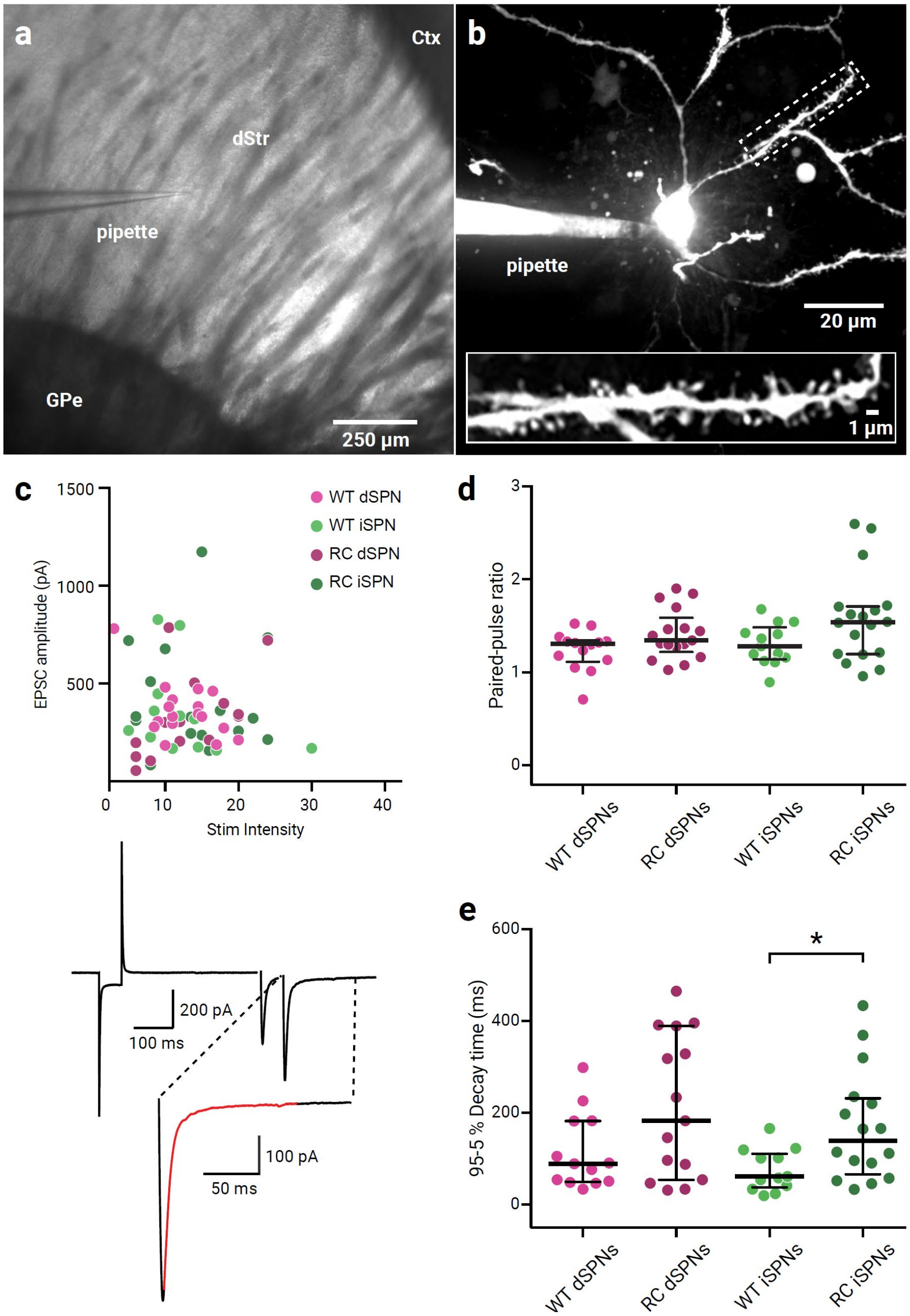

Supplementary Figure 5. Paired-pulse ratio and 95%–5% EPSC decay time analysis of corticostriatal input. a. Representative brightfield photomicrograph of a voltage-clamp recording of a WT dSPN in a parasagittal slice. GPe, external globus pallidus. **b**. Magnified fluorescent photomicrograph of the recorded dSPN in panel **a**. Pipette loading of Alexa Fluor 647 for live cell visualization was performed with confocal imaging. The identity of SPNs were confirmed by somatic morphology and high density of dendritic spines, characteristic of SPNs. Inset: magnified view of the boxed region showing a dendritic segment that is heavily decorated with spines. **c**. *Top*: Scatter plot of average first excitatory postsynaptic currents (EPSC) amplitudes per cell versus stimulation intensities showing majority of responses lie between 200–500 pA. *Bottom*: Mean voltage-clamp recordings of the dSPN shown in b. Paired-pulse stimulation of the cortex at

20 Hz elicited a pair of EPSCs. *Below mean trace*: Magnified view of the second EPSC amplitude and decay. The red portion depicts the 95%–5% decay time. **d**. Population data of paired-pulse ratios (PPR) of SPNs from WT and RC mice. No differences were found in dSPNs (PPR_{WT} = 1.31 ± 0.10, n = 14 cells; PPR_{RC} = 1.33 ± 0.15, n = 17 cells; p = 0.18, Mann–Whitney U test). Similarly, there were no changes in iSPNs (PPR_{WT} = 1.28 ± 0.14, n = 13 cells; PPR_{RC} = 1.54 ± 0.25, n = 18 cells; p = 0.11, Mann–Whitney U test). **e**. Population data of the 95%–5% EPSC decay times of SPNs from age-matched WT and RC mice. An increase in the 95%–5% decay times in the iSPNs of RC mice compared to WT was found ($Decay_{WT}$ = 61.1 ± 38.7 ms, n = 13 cells; $Decay_{RC}$ = 139.5 ± 81.1 ms, n = 18 cells; p = 0.036; Mann–Whitney U test). For dSPNs, no differences were found ($Decay_{WT}$ = 88.7 ± 40.3 ms, n = 14 cells; $Decay_{RC}$ = 182.9 ± 136.0 ms, n = 17 cells; p = 0.13, Mann–Whitney U test). * denotes $p < 0.05$. See Supplementary Table 6 for complete sample sizes and statistical results.

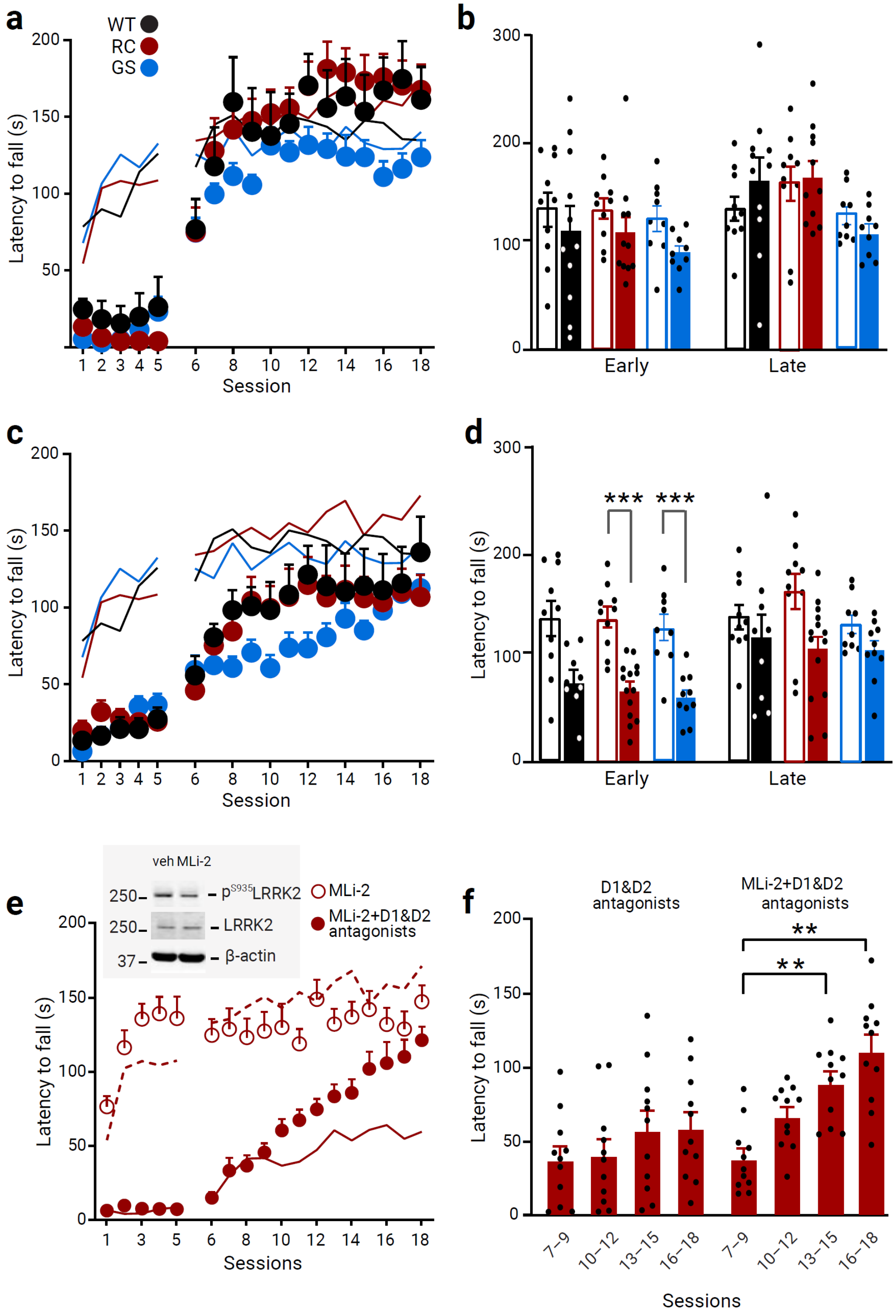

Supplementary figure 6. Effect of D1 or D2 antagonism on dopamine-dependent motor learning. Rotarod performance of WT, RC, and GS mice that were administered antagonists for either D1 (SCH23390) (**a–b**) or D2 receptors (eticlopride) (**c–d**), 30 min prior to training for the first five days. Solid traces are saline control plotted in Figure 3b for reference. **b** and **d**. Averaged latency of saline control (open bars) and drug-treated mice (filled bars). Early and late refer to sessions 6–8 and 16–18, respectively, of the no drug recovery phase (D1R antagonist treated

groups: n_{WT} = 11, n_{RC} = 11, and n_{GS} = 9 mice; D2R antagonist treated groups: n_{WT} = 9, n_{RC} = 14, and n_{GS} = 10 mice; *** $p < 0.001$ vs genotype-matched saline control). **e–f**. The motor learning deficit induced by dopamine antagonism was not observed in RC mice pre-treated with the LRRK2 inhibitor MLi-2 (5mg/Kg), which was administered daily, 60 min prior to training throughout the rotarod task (sessions 1-18). MLi-2 treated mice were coadministered with either saline (open circles) or antagonist cocktail (closed circles). Dash and solid lines, respectively, represent the saline control or dopamine receptor antagonist treated groups. Inset shows a decrease in S935 LRRK2 phosphorylation, reflecting LRRK2 kinase activity in WT mice after 60 min MLi-2 administration. **f**. Average latency in blocks of 3 sessions during the drug-free phase from **e**. RC with D1+D2 antagonists: n = 11 mice; MLi-2 with D1+D2 antagonists: n = 11 mice; ** $p < 0.01$).

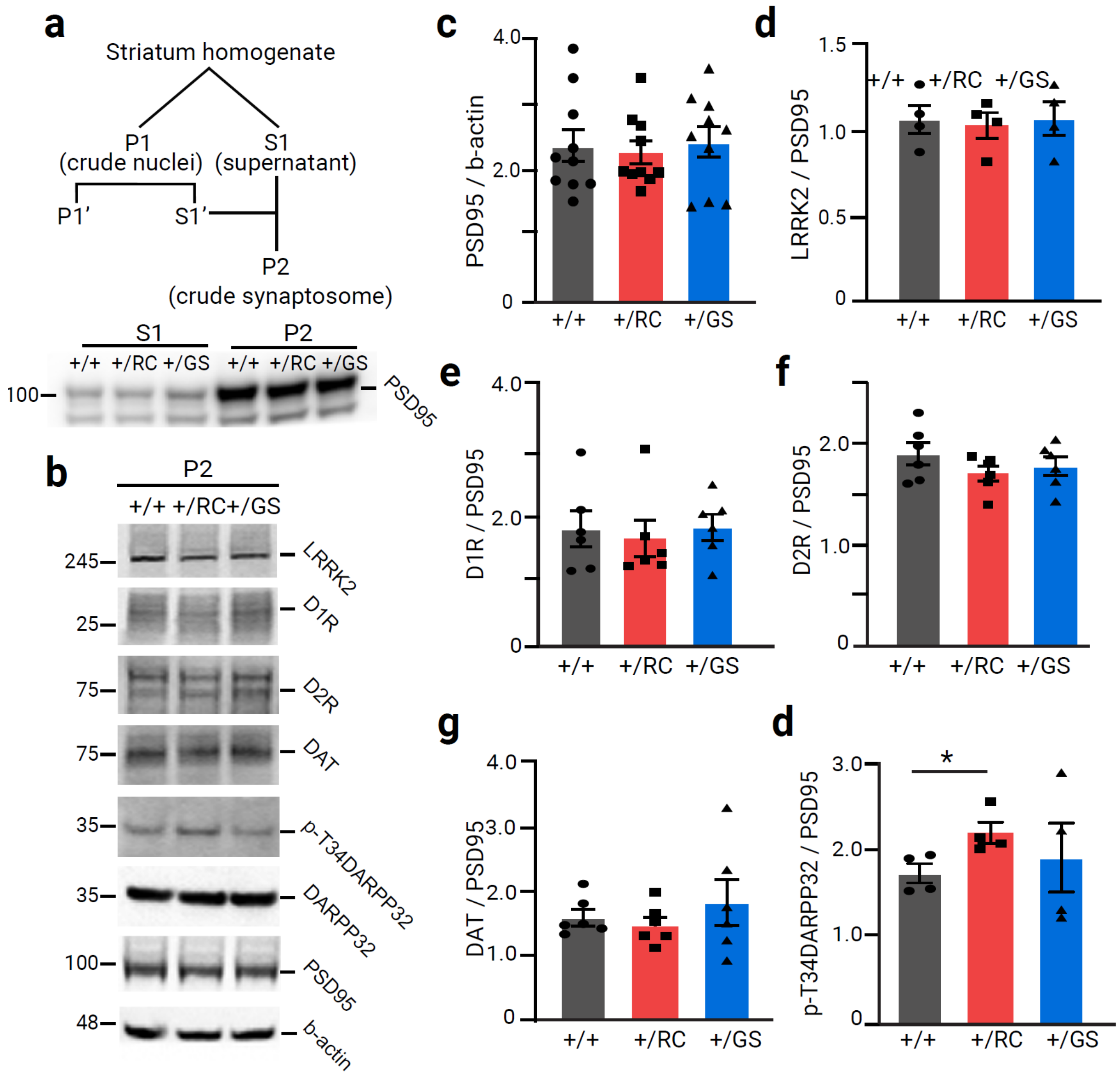

Supplementary Figure 7. Dopamine signaling component levels across LRRK2 mutations. **a**. Workflow schematic for striatal extract subcellular fractionation method. P2 (crude synaptosomal) fraction was used in the following WB experiments. WB shows the relative enrichment of PSD95 in the P2 fractions **b**. Representative WB analysis of WT, RC, and GS P2 fractions probed for LRRK2, D1R, D2R, DAT, p-T34-DARPP32 and DARPP32, PSD95, and actin, as shown. **c–g**. Quantification of PSD95 normalized to β-actin shows no differences in PSD95 expression across genotypes. LRRK2, D1R, D2R, DAT, and p-T34-DARPP32 normalized to PSD95. Summary graphs reflect the mean and error bars reflect SEM, * $p < 0.05$, unpaired t-test (n = 5–10).

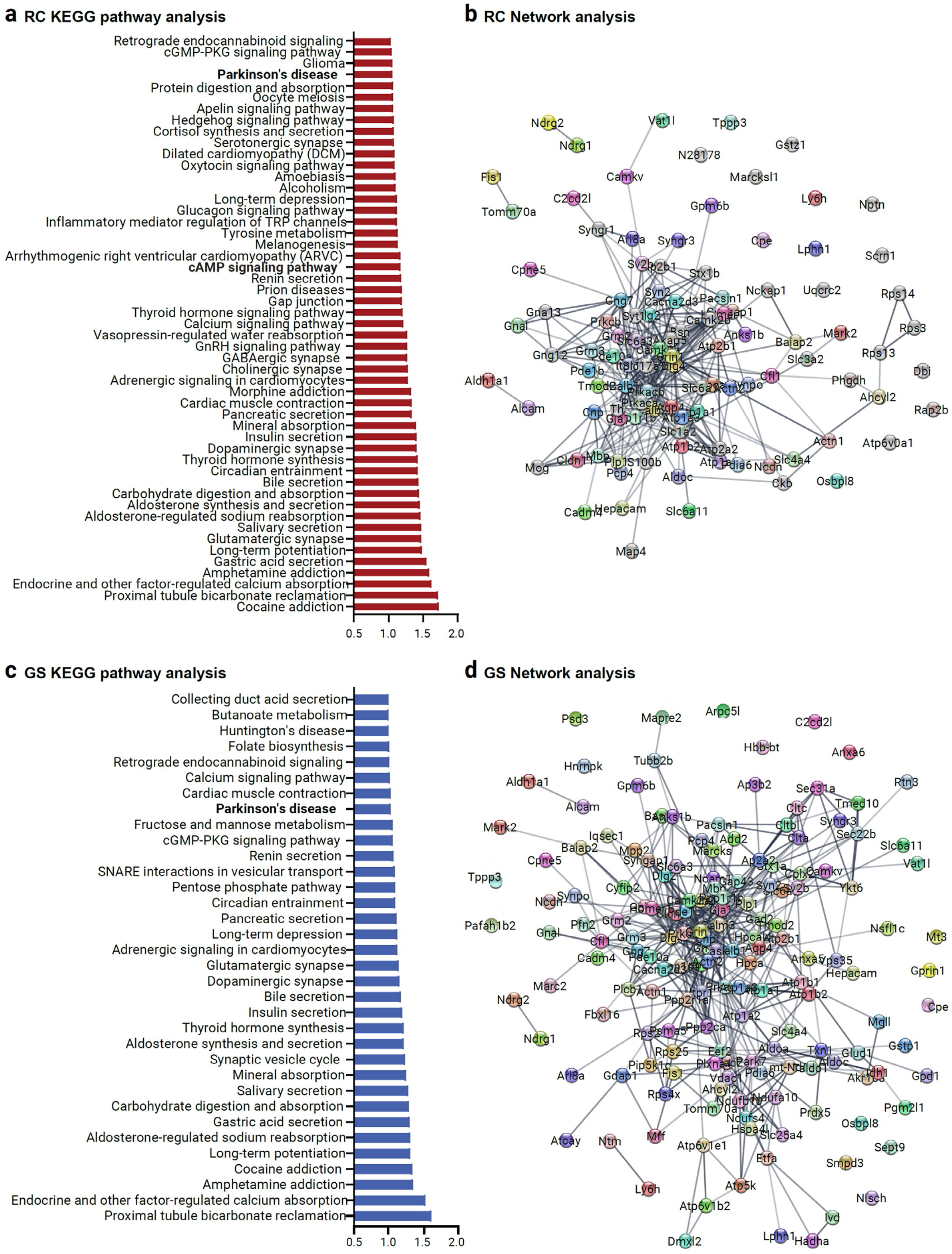

Supplementary Figure 8. Pathway and network analysis of the differentially expressed proteins in the RC and GS rotarod trained mice.

KEGG pathway (**a, c**) and network (**b, d**) analysis of top enriched pathways in RC and GS versus WT pairwise comparisons. KEGG annotated pathways with strength (log10 observed/expected) ≥ 1 were considered the top altered annotated pathways in the pairwise comparisons.

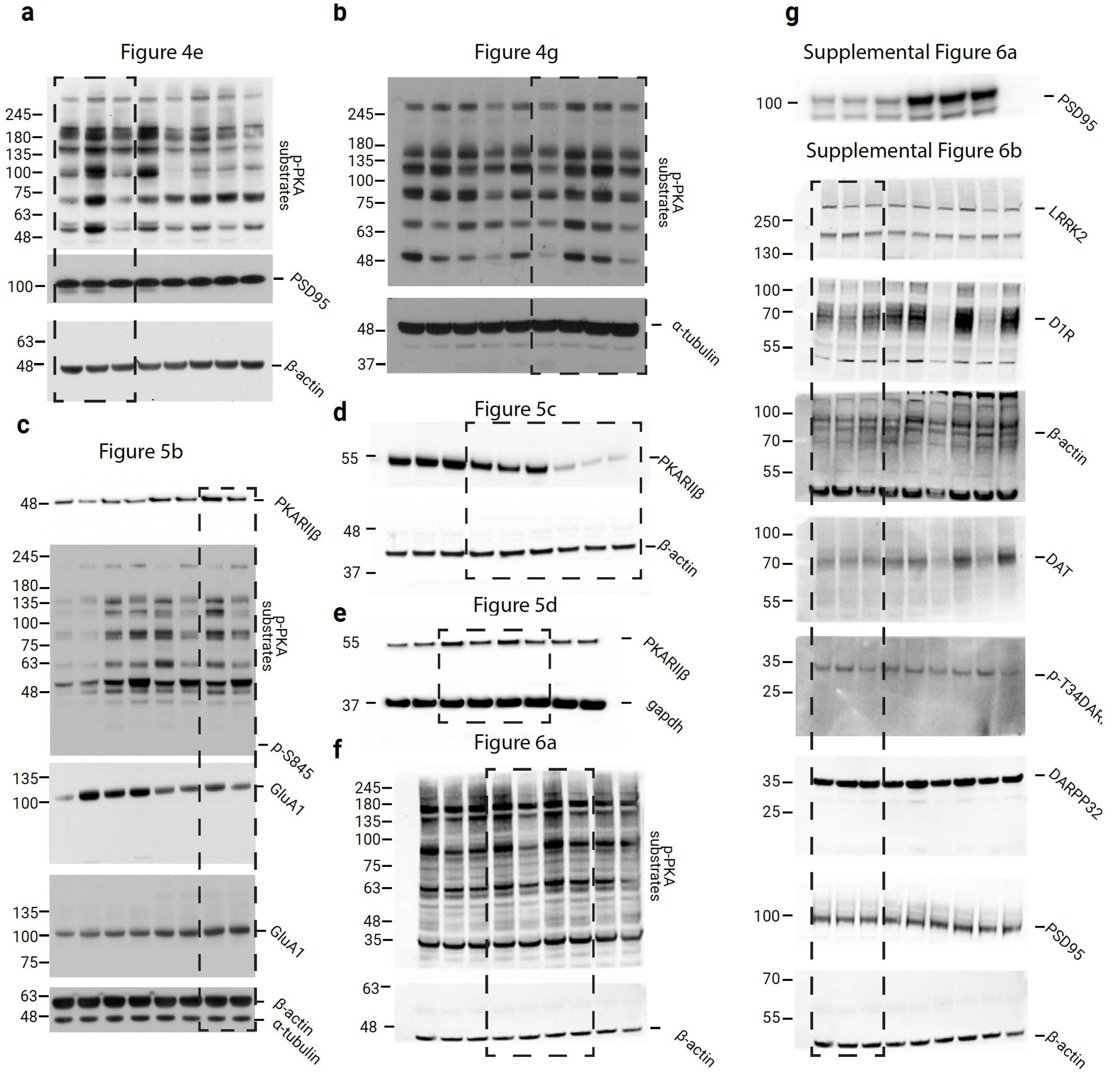

Supplementary Figure 9. **Unprocessed scans of Western blots. a-g.** The Figure panel where the corresponding Western blots are found is indicated on the top of each gel. The dashed box indicates the area of the scan presented in the relevant Figures.

Supplementary Table 1. Summary of electrically-evoked dopamine release

	Median ± MAD (nM)	n (measures)	n (mice by sex)	$p^{\dagger}$
WT	1058 ± 118	10	7(m); 3(f)	–
RC	539 ± 143	9	4(m); 5(f)	**0.006**†
GS	555 ± 218	13	5(m); 8(f)	**0.001**†

†Mann-Whitney U test compared to wild type values.

SupplementaryTable 2. Half-maximum and maximal F-I difference summary

R1441C half-maximum firing values

		n (mice)	n (slices)	dSPN (I = 475 pA) Median ± MAD	n (neurons)	$p^{\dagger}$	iSPN(I = 325 pA) Median ± MAD	n (neurons)	$p^{\dagger}$
Half-max firing (spikes)	WT	10	16	12 ± 4	20	0.2067	14 ± 5	17	**0.037**
	RC	14	19	14 ± 3	30		6 ± 6	18	

The cited currents are for where the half-maximal firing response occurred in WT mice and used to compare the corresponding firing response in mutant mice. †Mann-Whitney U test; boldface indicates significance, $p < 0.05$.

R1441C maximal difference of iSPN firing values

		n (mice)	n (slices)	iSPN (I = 625 PA) Median ± MAD	n (neurons)	$p^{\dagger}$
firing at max diff (spikes)	WT	10	16	22.0 ± 4.5	17	**0.0011**
	RC	14	19	15.0 ± 5.0	23	

The cited current is for where the maximal difference in firing of iSPNs occurred between WT and RC mice. †Mann-Whitney U test; boldface indicates significance, $p < 0.05$.

G2019S half-maximum firing values

		n (mice)	n (slices)	dSPN (I = 375 pA) Median ± MAD	n (neurons)	$p^{\dagger}$	iSPN (I = 325 pA) Median ± MAD	n (neurons)	$p^{\dagger}$
Half-max firing (spikes)	WT	8	12	22 ± 4	13	0.1229	20 ± 4	20	0.6590
	GS	12	23	23 ± 2	29		23 ± 3	28	

The cited currents are for where the half-maximal firing response occurred in WT mice and used to compare the corresponding firing response in mutant mice. †Mann-Whitney U test

Supplementary Table 3. Summary of maximal firing across sweeps

R1441C maximal firing summary

		n (mice)	n (slices)	dSPN Median ± MAD	dSPN n (neurons)	dSPN $p^{\dagger}$	iSPN Median ± MAD	iSPN n (neurons)	iSPN $p^{\dagger}$
maximal firing (spikes)	WT	10	16	18.5 ± 2	10	0.2200	23 ± 4.5	16	**0.0114**
	RC	14	19	22 ± 2	22		18.5 ± 3.5	16	

†Mann-Whitney U test; boldface indicates significance, $p < 0.05$.

R1441C current values for maximal firing summary

		n (mice)	n (slices)	dSPN Median ± MAD	dSPN n (neurons)	dSPN $p^{\dagger}$	iSPN Median ± MAD	iSPN n (neurons)	iSPN $p^{\dagger}$
current for maximal firing (pA)	WT	10	16	587.5 ± 87.5	10	0.8022	525 ± 125	16	0.4607
	RC	14	19	525 ± 125	19		500 ± 100	16	

†Mann-Whitney U test.

G2019S maximal firing summary

		n (mice)	n (slices)	dSPN Median ± MAD	dSPN n (neurons)	dSPN $p^{\dagger}$	iSPN Median ± MAD	iSPN n (neurons)	iSPN $p^{\dagger}$
maximal firing (spikes)	WT	8	16	21 ± 3	10	0.6606	19 ± 3	17	0.0835
	RC	12	18	22 ± 2	18		22 ± 3	24	

†Mann-Whitney U test.

G2019S current values for maximal firing summary

		n (mice)	n (slices)	dSPN Median ± MAD	dSPN n (neurons)	dSPN $p^{\dagger}$	iSPN Median ± MAD	iSPN n (neurons)	iSPN $p^{\dagger}$
7current for maximal firing (pA)	WT	8	16	660 ± 50	10	0.9898	550 ± 75	17	0.9633
	RC	12	19	637.5 ± 75	18		525 ± 75	24	

†Mann-Whitney U test.

Supplementary Table 4. General membrane electrophysiological properties

R1441C general membrane electrophysiological properties

		n (mice)	n (slices)	dSPN Median ± MAD	dSPN n (neurons)	dSPN $p^{\dagger}$	iSPN Median ± MAD	iSPN n (neurons)	iSPN $p^{\dagger}$
Rheobase (pA)	WT	10	16	300 ± 50	21	0.0850	225 ± 75	20	0.4348
	RC	14	19	300 ± 50	31		275 ± 125	23	
Cm (pF)	WT	10	16	78.33 ± 15.59	25	0.2333	68.83 ± 13.89	27	**0.0388**
	RC	14	19	81.27 ± 14.41	38		87.38 ± 13.34	27	
Rin (MΩ)	WT	10	16	56.13 ± 18.26	17	0.2333	67.75 ± 21.05	17	0.1409
	RC	14	19	69.47 ± 17.03	21		85.03 ±18.54	14	
Vm (MΩ)	WT	10	16	−90.52 ± 1.58	17	0.2333	−89.61 ± 2.35	16	0.4904
	RC	14	19	−90.85 ± 2.96	21		−89.74 ± 1.51	15	

†Mann-Whitney U test; boldface indicates significance, $p < 0.05$. Cm, membrane capacitance; Rin, input resistance; Vm, membrane potential.

G2019S general membrane electrophysiological properties

		n (mice)	n (slices)	dSPN Median ± MAD	dSPN n (neurons)	dSPN $p^{\dagger}$	iSPN Median ± MAD	iSPN n (neurons)	iSPN $p^{\dagger}$
Rheobase (pA)	WT	8	12	250.00 ± 25.00	13	**0.0078**	162.50 ± 87.50	20	**0.0046**
	GS	12	23	325.00 ± 50.00	29		225.00 ± 50.00	28	
Cm (pF)	WT	8	12	88.12 ± 10.81	15	0.2855	74.65 ± 12.26	19	0.5085
	GS	12	23	95.37 ± 12.03	27		82.66 ± 15.25	30	
Rin (MΩ)	WT	8	12	50.01 ± 4.39	13	0.4203	52.19 ± 10.87	20	0.5499
	GS	12	23	40.66 ± 11.14	19		52.84 ± 12.07	19	
Vm (MΩ)	WT	8	12	−87.59 ± 3.42	12	0.5496	−86.21 ± 3.94	19	0.0849
	GS	12	23	−87.73 ± 3.21	29		−89.62 ± 3.36	28	

†Mann-Whitney U test; boldface indicates significance, $p < 0.05$. Cm, membrane capacitance; Rin, input resistance; Vm, membrane potential.

Supplementary Table 5. Action potential characteristics

R1441C action potential characteristics

		n (mice)	n (slices)	dSPN Median ± MAD	dSPN n (neurons)	dSPN $p^{\dagger}$	iSPN Median ± MAD	iSPN n (neurons)	iSPN $p^{\dagger}$
Threshold (mV)	WT	10	16	−47.78 ±5.08	17	0.7804	−46.36 ± 2.63	22	0.4348
	RC	14	19	−46.10 ± 3.00	20		−49.69 ± 2.89	19	
ISI (ms)	WT	10	16	10.20 ± 2.00	17	0.7189	8.00 ± 1.00	17	**0.0388**
	RC	14	19	10.20 ± 1.40	24		9.40 ± 1.20	19	
FWHM (ms)	WT	10	16	1.53 ± 0.19	17	0.3602	1.54 ± 0.144	16	0.1409
	RC	14	19	1.63 ± 0.12	25		1.72 ± 0.11	19	
dV/dt (mV/ms)	WT	10	16	50.90 ± 5.06	17	0.2167	48.73 ± 3.68	16	0.4904
	RC	14	19	45.50 ± 3.33	25		49.78 ± 2.98	19	

†Mann-Whitney U test; boldface indicates significance, $p < 0.05$. ISI, interspike interval; FWHM, full width at half maximum.

G2019S action potential characteristics

		n (mice)	n (slices)	dSPN Median ± MAD	dSPN n (neurons)	dSPN $p^{\dagger}$	iSPN Median ± MAD	iSPN n (neurons)	iSPN $p^{\dagger}$
Threshold (mV)	WT	8	12	−44.23 ±2.95	13	0.8126	−43.94 ±4.07	20	0.1004
	GS	9	14	−44.21 ± 3.57	19		−46.42 ±13.59	22	
ISI (ms)	WT	8	12	8.90 ± 1.60	13	0.7017	8.95 ± 1.85	20	**0.0401**
	GS	9	14	7.70 ± 0.90	29		7.15 ± 1.25	28	
FWHM (ms)	WT	8	12	1.61 ± 0.12	13	0.1016	1.60 ± 0.16	20	**0.0039**
	GS	9	14	1.40 ± 0.14	29		1.41 ±0.07	28	
dV/dt (mV/ms)	WT	8	12	43.89 ± 4.54	13	0.1270	44.45 ± 5.12	17	0.1383
	GS	9	14	49.80 ± 6.03	29		51.38 ± 7.13	28	

†Mann-Whitney U test; boldface indicates significance, $p < 0.05$. ISI, interspike interval; FWHM, full width at half maximum.

Supplementary Table 6. Summary of RC iSPN paired-pulse ratio and 95%–5% EPSC decay time

R1441C paired-pulse ration

		n (mice)	n (slices)	dSPN Median ± MAD	dSPN n (neurons)	dSPN p[†]	iSPN Median ± MAD	iSPN n (neurons)	iSPN p[†]
paired-pulse ratio	WT	8	9	1.31 ± 0.10	14	0.18	1.28 ± 0.14	13	0.11
	RC	12	15	1.33 ± 0.15	17		1.54 ± 0.25	18	

[†]Mann-Whitney U test.

R1441C 95%–5% decay time

		n (mice)	n (slices)	dSPN Median ± MAD	dSPN n (neurons)	dSPN p[†]	iSPN Median ± MAD	iSPN n (neurons)	iSPN p[†]
95%–5% decay time (ms)	WT	8	9	88.7 ± 40.3	14	0.13	61.1 ± 38.7	13	**0.036**
	RC	12	15	182.9 ± 136	17		139.5 ± 81	18	

[†]Mann-Whitney U test; boldface indicates significance, $p < 0.05$.

Supplementary Table 7. Motor learning Summary

Early sessions 6–8

	WT		RC		GS	
	n (mice)	$p^{\dagger}$	n (mice)	$p^{\dagger}$	n (mice)	$p^{\dagger}$
Saline	10		10		9	
Saline vs. D1 + D2 antagonism	10, 13	< 0.0001	10, 11	< 0.0001	9, 11	0.0007
Saline vs. D1 antagonism	10, 11	0.9913	10, 11	0.9593	9, 9	0.7990
Saline vs. D2 antagonism	10, 9	0.0549	10, 14	0.0190	9, 10	0.0128

	Saline		D1 + D2 antagonism		D1 antagonism			D2 antagonism	
	n (mice)	$p^{\dagger}$	n (mice)	$p^{\dagger}$	n (mice)	n (mice)	$p^{\dagger}$	n (mice)	$p^{\dagger}$
WT vs. RC	10, 10	1.0000	13, 11	0.9908	11, 11		1.0000	9, 14	1.0000
WT vs. GS	10, 9	1.0000	13, 11	1.0000	11, 9		0.9781	9, 10	0.9981
RC vs. GS	10, 9	1.0000	11, 11	0.9908	11, 9		0.9936	14,10	1.0000

Late sessions 16–18

	WT		RC		GS	
	n (mice)	$p^{\dagger}$	n (mice)	$p^{\dagger}$	n (mice)	$p^{\dagger}$
Saline vs. D1 + D2 antagonism	13	0.9999	11	0.0001	11	0.9842
Saline vs. D1 antagonism	11	0.5480	11	1.0000	9	0.9998
Saline vs. D2 antagonism	9	1.0000	14	0.1492	10	0.9904

	Saline		D1 + D2 antagonism		D1 antagonism		D2 antagonism	
	n (mice)	$p^{\dagger}$	n (mice)	$p^{\dagger}$	n (mice)	$p^{\dagger}$	n (mice)	$p^{\dagger}$
WT vs. RC	10,10	0.989	13, 11	0.0429	11 ,11	1.0000	9, 14	1.0000
WT vs. GS	10, 9	1.0000	13, 11	0.0952	11, 9	0.3641	9, 10	1.0000
RC vs. GS	10, 9	1.0000	11, 11	0.4980	11, 9	0.2661	14, 10	1.0000
