# Supplementary material for: R1441C and G2019S LRRK2 knockin mice have distinct striatal molecular, physiological, and behavioral alterations
Source: Commun Biol. 2022 Nov 10;5:1211. doi: 10.1038/s42003-022-04136-8 (PMC9649688; doi:10.1038/s42003-022-04136-8)
Supplement: Supplementary file 3 — Description of Additional Supplementary Files [file 42003_2022_4136_MOESM3_ESM.pdf]

# Description of Additional Supplementary Files

**File name:** Supplementary Data 1
**Description:** Full list of altered proteins and pathways in RC vs. WT mice.

**File name:** Supplementary Data 2
**Description:** Full list of altered proteins and pathways in GS vs. WT mice.

**File name:** Supplementary Data 3
**Description:** Source data of Figures 3 b,c,d,e.

**File name:** Supplementary Data 4
**Description:** Source data of Figures 4 f,h.

**File name:** Supplementary Data 5
**Description:** Source data of Figures 5 c,f.

**File name:** Supplementary Data 6
**Description:** Source data of Figures 6b,c,d,e,f.
