# Supplementary material for: R1441C and G2019S LRRK2 knockin mice have distinct striatal molecular, physiological, and behavioral alterations
Source: Commun Biol. 2022 Nov 10;5:1211. doi: 10.1038/s42003-022-04136-8 (PMC9649688; doi:10.1038/s42003-022-04136-8)
Supplement: Supplementary file 10 — Reporting Summary [file 42003_2022_4136_MOESM10_ESM.pdf]

nature research

Corresponding author(s): LOUKIA PARISIADOU

Last updated by author(s): Oct 7, 2022

Reporting Summary

Nature Research wishes to improve the reproducibility of the work that we publish. This form provides structure for consistency and transparency in reporting. For further information on Nature Research policies, see our Editorial Policies and the Editorial Policy Checklist.

Statistics

For all statistical analyses, confirm that the following items are present in the figure legend, table legend, main text, or Methods section.

n/a	Confirmed	
☐	☒	The exact sample size (n) for each experimental group/condition, given as a discrete number and unit of measurement
☐	☒	A statement on whether measurements were taken from distinct samples or whether the same sample was measured repeatedly
☐	☒	The statistical test(s) used AND whether they are one- or two-sided<br>*Only common tests should be described solely by name; describe more complex techniques in the Methods section.*
☒	☐	A description of all covariates tested
☐	☒	A description of any assumptions or corrections, such as tests of normality and adjustment for multiple comparisons
☐	☒	A full description of the statistical parameters including central tendency (e.g. means) or other basic estimates (e.g. regression coefficient) AND variation (e.g. standard deviation) or associated estimates of uncertainty (e.g. confidence intervals)
☐	☒	For null hypothesis testing, the test statistic (e.g. F, t, r) with confidence intervals, effect sizes, degrees of freedom and P value noted<br>*Give P values as exact values whenever suitable.*
☒	☐	For Bayesian analysis, information on the choice of priors and Markov chain Monte Carlo settings
☒	☐	For hierarchical and complex designs, identification of the appropriate level for tests and full reporting of outcomes
☒	☐	Estimates of effect sizes (e.g. Cohen's d, Pearson's r), indicating how they were calculated

Our web collection on statistics for biologists contains articles on many of the points above.

Software and code

Policy information about availability of computer code

Data collection	Electrophysical and electrochemical data was acquired on pClamp.
Data analysis	Analysis and visual representations were done using SAS, Prism, and MATLAB.

For manuscripts utilizing custom algorithms or software that are central to the research but not yet described in published literature, software must be made available to editors and reviewers. We strongly encourage code deposition in a community repository (e.g. GitHub). See the Nature Research guidelines for submitting code & software for further information.

Data

Policy information about availability of data

All manuscripts must include a data availability statement. This statement should provide the following information, where applicable:

- Accession codes, unique identifiers, or web links for publicly available datasets
- A list of figures that have associated raw data
- A description of any restrictions on data availability

Codes are openly available at Zenodo, DOIs:10.5281/zenodo.7153493; 10.5281/zenodo.7153493; 10.5281.7153540
The mass spectrometry proteomics data have been deposited to the ProteomeXchange Consortium via the PRIDE partner repository with the dataset identifier PXD037003. Raw tabular data of electrophysiology, FSCV, and behavioral tests are available through the open access option on the Zenodo data repository site with the following DOIs (10.5281/zenodo.7153552, and 10.5281/zenodo.7153453).
The fast-scanning voltammetry, ex vivo physiology, behavioral data and the intracranial injections are deposited in protocols.io

Field-specific reporting

Please select the one below that is the best fit for your research. If you are not sure, read the appropriate sections before making your selection.

☒ Life sciences ☐ Behavioural & social sciences ☐ Ecological, evolutionary & environmental sciences

For a reference copy of the document with all sections, see nature.com/documents/nr-reporting-summary-flat.pdf

Life sciences study design

All studies must disclose on these points even when the disclosure is negative.

Sample size	Sample size was determined using G*Power (https://www.psychologie.hhu.de).
Data exclusions	Data recordings for electrophysiology were excluded if the access resistance deviated more than 20% from baseline. Similarly, for voltammetric recordings, if post experimental electrode sensitivity deviated by 10% from pre-experimental calibration done daily a the start of each experiment. For behavioral data, a subject would be excluded if its health condition was determined inappropriate for behavioral testing by certified veterinarians and the experimenters.
Replication	Data replication was done with a minimum of six mice per experimental group except for some WB experiments. For physiology, experiments were done from multiple mice per age-matched groups. Recording data were from multiple site of multiple slices from each mouse. Sample size of recordings and mice are all explicitly stated.
Randomization	Age-matched mice were randomly selected for experimental procedures.
Blinding	Experimenters were kept double blinded for all experiments.

Reporting for specific materials, systems and methods

We require information from authors about some types of materials, experimental systems and methods used in many studies. Here, indicate whether each material, system or method listed is relevant to your study. If you are not sure if a list item applies to your research, read the appropriate section before selecting a response.

Materials & experimental systems

n/a	Involved in the study	
☐	☒	Antibodies
☒	☐	Eukaryotic cell lines
☒	☐	Palaeontology and archaeology
☐	☒	Animals and other organisms
☒	☐	Human research participants
☒	☐	Clinical data
☒	☐	Dual use research of concern

Methods

n/a	Involved in the study	
☒	☐	ChIP-seq
☒	☐	Flow cytometry
☒	☐	MRI-based neuroimaging

Antibodies

Antibodies used	Primary antibodies specific for pS935 LRRK2 (Abcam Cat# ab230261, RRID:AB_2811274, 1:1000), total LRRK2 (Abcam Cat# ab133474, RRID:AB_2713963, 1:1000), and β-actin (Sigma-Aldrich Cat# A1978, RRID:AB_476692, 1:3000), Scientific MA1-045, 1:3000), dopamine D1R (Sigma-Aldrich Cat# D187, RRID:AB_1840789), dopamine D2R (Frontier Institute Cat# D2R-Rb, RRID:AB_2571596), DAT (Millipore Cat# MAB368, RRID:AB_94947), p-T74DARPP-32 (Cell Signaling Technology Cat# 12438, RRID:AB_2797914), total DARPP-32 (Cell Signaling Technology Cat# 2306, RRID:AB_823479), PSD95 (Thermo Fisher Scientific Cat# MA1-045, RRID:AB_325399, 1:1000), p-PKA substrates (Cell Signaling Technology Cat# 9624, RRID:AB_331817) were used
Validation	pS935, ab230261. This antibody was developed with the support of The Michael J. Fox Foundation (MJFF) and in partnership with Dr. Dario Alessi (MRC Protein Phosphorylation Unit, University of Dundee) and has been validated by many research groups using diverse LRRK2 inhibitors, total LRRK2, ab133476 KO validated https://www.abcam.com/lrrk2-antibody-mjff4-c81-8-ab133476.html Dopamine D1R and D2R were validated in KO mice and all the rest of the antibodies were validated either with mRNA or KO tissues in previous studies.

Animals and other organisms

Policy information about studies involving animals; ARRIVE guidelines recommended for reporting animal research

Laboratory animals	LRR2R1441C and LRRK2G2019S, Drd1-dTomato and Drd2-eGFP BAC transgenic mice

Wild animals	n/a
Field-collected samples	n/a
Ethics oversight	All procedures were done in accordance with Northwestern University Animal Care and Use Committee guidelines.

Note that full information on the approval of the study protocol must also be provided in the manuscript.
